# Some fundamental insights into biological redox catalysis from the electrochemical characteristics of enzymes attached directly to electrodes

**DOI:** 10.1016/j.electacta.2021.138836

**Published:** 2021-09-10

**Authors:** Fraser A. Armstrong

**Affiliations:** Department of Chemistry, University of Oxford, Oxford OX1 3QR, UK

## Abstract

This article outlines examples of where electrochemical investigations of electrocatalysis by proteins immobilised on an electrode reveal fundamental information about electron-proton coupling in catalysis and provide a new way to energise, control and observe multi-enzyme cascades.

## 1 Introduction

Traditional electrochemical textbooks and courses focus heavily on the role of diffusion in facilitating electron-transfer (ET) reactions at electrodes. Accordingly, most molecular electrocatalysts that have been studied in detail are freely diffusing complexes, the field being pioneered and placed on a sound analytical basis by Saveant and others [1]. By 1990, interest in small-protein electrochemistry was maturing and many had realised that enzymes needed to be adsorbed on an electrode in order to drive and observe their catalytic reactions. Enzymes are large, pre-assembled and largely self-contained catalysts: thanks to modern synchrotron methods, their static structures are known to high precision many nanometers out from the site at which the bond-changing catalytic events occur, while molecular dynamics calculations add the possibility of predicting transient states.

In this article, I have endeavoured to identify a common theme relating to why protein molecules undertaking kinetically-limited *coupled* electron-transfer reactions need to be attached to an electrode and why redox enzymes cannot be successfully understood in the same way as small, mobile molecular electrocatalysts. I have focused entirely on redox reactions that are not mediated by small electron mediators, electron transfer instead being ‘direct’. Mediators are essential in applications such as biosensors, as they extend the range of enzymes that can be used and yield stable, robust devices with large catalytic currents [2], [3], [4], [5]. However, as mentioned later, use of mediators may mask fundamental characteristics of enzymes that electrochemical techniques are otherwise so powerful in revealing.

Unfortunately, dynamic electrochemistry is not widely appreciated by biochemistry and even chemistry communities, and there is still a reluctance to accept it as a unique tool in enzymology. There are so many different equations and often there are too many variables to arrive at firm conclusions. Even the concept of reaction rate is often confused, an example being that in steady-state electrocatalysis, the constant current itself is a measure of turnover rate, while the rate of change of current corresponds to the rate of activation or deactivation. To demonstrate the transparent approach, I have outlined a handful of ‘stand-alone’ observations, immediately visible by eye, that require little further processing to extract information that is otherwise invisible or intractable. Some of these simple conclusions have fundamental significance.

## 2 What does a layer of protein molecules on an electrode look like ?

In the early days of bioelectrochemistry, adsorption of proteins on an electrode tended to be discussed in the negative context of electrode fouling- protein molecules having a natural tendency to accumulate at interfaces. The issue of whether or not proteins unfold or retain their native structure in the adsorbed state was considered in detail by Norde and Lyklema, who had carried out extensive studies on the binding of various proteins to surfaces with and without unfolding [6].

**Fig. 1:**
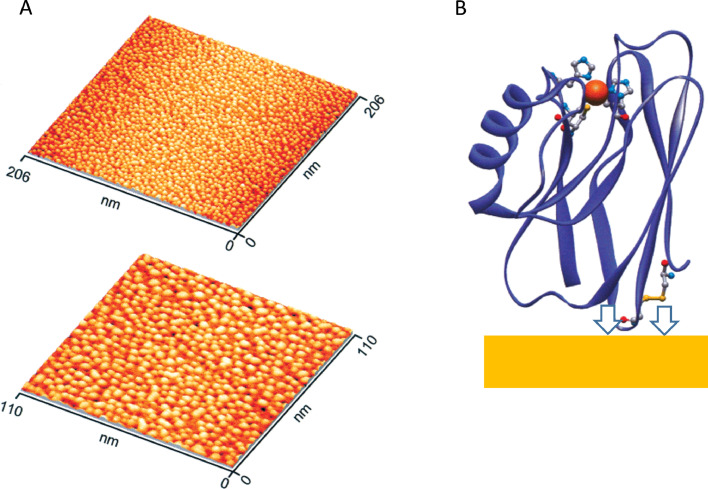
**A.** In situ STM images of azurin molecules adsorbed at a Au(111) surface obtained in 50 mM ammonium acetate (pH 4.6) projected at different scales. **B.** representation of how azurin molecules are covalently attached to the Au surface, using two cysteine-S atoms released by reduction of a cysteine –S-S- pair. Adapted with permission from American Chemical Society, Reference 7.

Professor Jens Ulstrup has been a leading advocate of the need to understand, at a detailed physical level, how an electron is transferred between an electrode and the active site in an adsorbed protein molecule. He recognised the importance of dealing with a highly uniform protein population or, even better, a single protein molecule. To achieve this goal, a protein molecule ideally needed to be attached to an atomically flat electrode, and uniformly orientated in way that would optimize electron tunnelling: Ulstrup introduced a precise term − protein monolayer voltammetry (PMV) [7,8]. Importantly, an atomically flat surface would also allow imaging by STM or AFM; hence it was important and reassuring to obtain a very clear picture (Fig. 1A) of a small ‘blue’ Cu protein, azurin (14 kDa) deposited across an atomically flat Au electrode, the molecular coverage being directly countable and amounting to about 10 pmol cm^−2^ [7]. This value gives an excellent guide as to what can be expected normally, the two obvious reality factors, (i) that practical electrode surfaces are normally not flat (increasing the area), and (ii) redox proteins are highly anisotropic and orientation sensitive (decreasing productive interactions) serving to compensate for each other.

The azurin molecules are covalently attached to the Au surface through a pair of neighbouring cysteine-S atoms (which normally form a cysteine-S-S crosslink) located on the opposite side of the molecule to the Cu site, leading to a measurable distance between electrode and Cu atom. (Fig. 1B). One of Ulstrup's aims in these experiments was to understand the medium dependence governing long-range ET between the electrode and redox-active sites in protein molecules. By having the protein immobilised on an electrode, it becomes possible to use fast scan cyclic voltammetry to analyse electron (and coupled proton transfer) kinetics, useful data being obtained at scan rates up to and exceeding 1000 V *s* ^−^ ^1^ [9,10]. In the case of the azurin monolayer on Au, cyclic voltammetry did not prove successful, but using electrochemical impedance spectroscopy (with ‘redox-silenced’ Zn azurin serving as a control) the standard (exchange) rate constant was determined to be 30 ± 5 *s* ^−^ ^1^, a substantial value given the long distance (approximately 25 Å between electrode and Cu atom) that is involved [7]. Ulstrup and coworkers extended their work to an enzyme, a blue Cu oxidase known as laccase, achieving highly active (sub)monolayers of laccases on an atomically flat Au surface modified with a various types of self-assembled alkylthiol monolayer [11] With these systems they were able to correlate results from STM imaging with kinetic data obtained for the four-electron O_2_ reduction reaction.

Despite the desire to arrange protein molecules in such an ideal way, their complex shapes and large size suggest that a rough electrode surface should have a natural advantage over one that is atomically flat, in terms of overall coverage, binding strength and optimizing the electron-transfer rate. A flat electrode surface can accommodate only a limited number of bonding contacts with surface groups of a folded protein molecule, whereas a rough surface can offer far more, greatly increasing the strength of binding and statistical possibility of engaging a suitable electron-transfer pathway. The difference can be illustrated by comparing how an egg rolls around chaotically when placed on a table but is securely constrained in an egg tray. One such rough surface is abraded graphite, particularly one known as pyrolytic graphite ‘edge’ – PGE (avoiding the misleading term ‘edge-plane) which also comprises numerous carbon-oxide functionalities. It is important to clarify the meaning of ‘orientation’. Does orientation assume an atomically flat surface at which all protein molecules can be referenced to a common direction, or does orientation apply also to a rough surface in which each protein is considered in its own local environment, maximizing the tunnelling rate by minimizing distance ? Protein molecules are dynamic, so freedom to explore different orientations while remaining tethered should be beneficial. Efforts to control orientation include modifying an electrode by attaching molecules having an affinity for a specific zone on the protein surface that is suited for fast electron tunnelling. Systemmatic, uniform orientation of enzymes may be achieved by derivatizing the electrode surface with substrate analogues able to target a particular site on the protein surface: such experiments have been performed with blue Cu oxidases [12].

Electron-transferring centres in proteins have evolved to have low reorganization energies, in terms of the contribution both from the inner coordination shell and the outer environment (as these centres tend to be shielded from solvent). Consequently, it is expected that protein orientation with respect to the local electrode surface may have little effect on reorganisation energy but be very important in modifying the distance across which the electron must tunnel [13]. A model proposed in 2002 dealt with how a dispersion of tunnelling distances due to inhomogeneous orientations would affect the shape of catalytic voltammograms [14]. Most significantly, dispersion amongst rates resulted in the catalytic current continuing to increase with potential well beyond the value at which a limiting plateau would be expected –as less-well orientated enzyme molecules requiring a greater overpotential contribute increasingly to the current.

## 3 An early demonstration that complex, multi-step redox reactions require immobilisation of the protein on an electrode

**Fig. 2:**
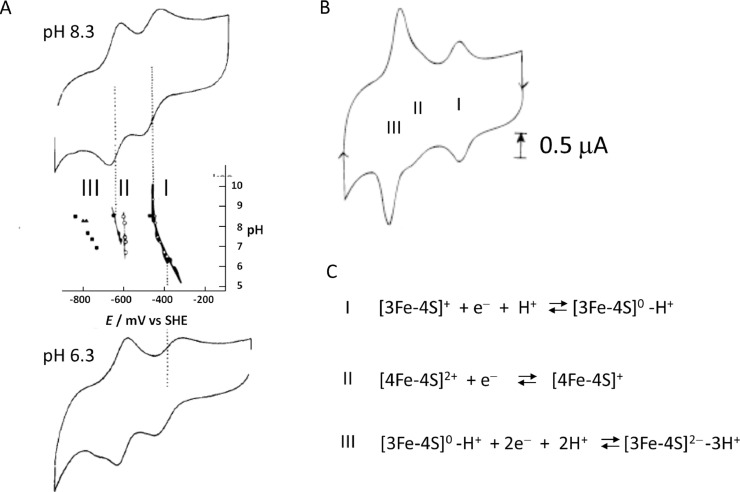
**A.** Cyclic voltamograms measured at pH 8.3 and 6.3 for a solution of the 7Fe ferredoxin from *Azotobacter chroococcum*, and the pH dependence of the [3Fe-4S]^+/0^ and [4Fe-4S]^2+/+^ redox couples (I and II, respectively) that appear as diffusion-controlled electron transfers. **B.** A cyclic voltammogram measured for a stable film of the ferredoxin without any additional protein in solution, revealing a third redox couple (III) that must be a copperative two-electron transfer **C.** Assignments of the three redox couples I, II and III.

Cooperative two-electron transfers (*n* = 2) in which the second electron transfer is spontaneous, are highly visible in cyclic voltammograms of immobilized systems. Ideally comprising oxidation and reduction peaks with small peak separations, the peak heights vary as n^2^ while widths vary as approximately 90/n mV at 25 °C, although each of these limiting cases will be eroded by dispersion of rates and potentials [9]. In earlier work on small electron-carrier [Fe-S] proteins known as ferredoxins, it was noted that an additional, unexpected redox couple appeared at more negative potential if a [3Fe-4S] cluster was present in the molecule [15]. The appearance of the new redox couple depended strongly on the protein being adsorbed on the electrode – there usually being little indication of its presence when the ferredoxin was in solution (Fig. 2A) [16]. With small, highly negatively charged proteins such as ferredoxins, polycations such as the antibiotic neomycin were found to greatly enhance adsorption at a PGE electrode, such an electrode being far removed from the atomically flat Au electrode preferred for surface layer characterization.

From the narrow shape and positions of the oxidation and reduction peaks for the additional couple, it was clear that the additional signal arose from a rapid cooperative multi-electron process and it was assigned to the [3Fe-4S]^0/2−^ couple, the peak areas being twice that assigned to the normal [3Fe-4S]^+/0^  couple (Fig. 2B) [16]. Both the normal and the new couple vanished if, in the case of certain proteins, the [3Fe-4S] cluster was transformed into a [M3Fe-4S]^2+/+^ cubane upon reductive uptake of an extraneous divalent metal cation (*M* = Fe, Zn, Cd) [17]. The redox couples for the 7Fe ferredoxin are explained in Fig. 2C. The appearance of the new narrow signal associated with couple III for all small proteins containing a [3Fe-4S] cluster showed that it must be inherent to this cluster type, and not an artefact (i.e. an unrecognised property of a particular protein). The coverage of the protein could easily be measured from the area (charge) contained within each peak: inspection over a range of values from 5 to 15 pmole cm^−2^ suggested that from a practical standpoint, signals due to one-electron transfers might become undetectable at a coverage below 2 pmole cm^−2^.

An obvious implication was that the reduced product was an all-Fe(II) cluster (since the fully oxidized [3Fe-4S]^+^ state contains three Fe(III) and one-electron reduction to the ‘0′ level was already accounted for at higher potential). Having established that a cooperative two-electron transfer is involved, it was expected that a charge compensation was necessary to avoid an unacceptable coulombic cost. The pH dependence for the new couple showed that at least one additional proton was bound; furthermore, a strong H/D solvent isotope effect appeared when CVs were measured at higher scan rates [18], and a mutant protein defective in proton delivery for the normal [3Fe-4S]^1/0^ couple showed a greatly suppressed signal amplitude [19]. The requirement for adsorption and the near-symmetrical form of the oxidation and reduction peaks in either direction suggested that the redox process was minimally of the ‘ECE’ type; where the chemical step ‘C’ is an intermediary proton-transfer reaction that neutralises the charge and may induce some overcompensating reorganisation that facilitates spontaneous transfer of the second electron, the complete sequence being a centrally-gated two-electron transfer. Such a complex process might not be completed upon simple diffusional encounter. In such a multi-step process, strong adsorption of the protein would offer a large advantage by indefinitely extending the encounter time, an important factor because protein molecules, being highly dynamic, may need to ‘visit’ numerous micro-conformations to complete the central coupling step.

In essence the ‘C’ step in the [3Fe-4S]^0/2−^ redox process is a proton transfer, but in the interests of retaining conventional nomenclature, ‘ECE’ will be taken to mean a core electron-proton-electron process. A second proton transfer can be included, but the gate separating first and second electron transfers remains the central coupling step. Protonation must occur at the Fe-S cluster itself, not on a nearby amino acid, because the ‘C’ signal varies little between different proteins, although it does appear as a diffusion-controlled reaction in one particular 7Fe ferredoxin, which led to partial characterisation of the reduced product [16]. Despite the very negative formal potential, no H_2_ is evolved, suggesting that protons are bound to S rather than Fe atoms (which would result in reactive hydridic-H). Later we will see how the ECE sequence translates rather impressively into electrocatalytic ‘hydride’ transfer by a special enzyme.

The ‘C’ redox couple is probably not important physiologically, and more detailed work was not pursued, but the marked appearance in the adsorbed state was mechanistically significant. The fact that a single [3Fe-4S] cluster displays two redox couples but with only one depending on the protein being adsorbed showed that immobilising a protein gives a special advantage for electron transfers that extend beyond the simple elementary level.

## 4 Extensions to enzymes and catalysis

Virtually all redox enzymes catalyse complex reactions that involve the transfer of two electrons and at least one proton at the active site. Many enzymes also require an activation step to enter the catalytic cycle, and it is difficult to see how they could be effective electrocatalysts unless anchored to an electrode. Enzymes that have evolved to perform long-range ET should always be amenable to study in the adsorbed state (given the right electrode) as at least one ET centre will lie close to the protein surface. Enzymes that evolved only to move electrons at and within an occluded active site and lacking an ET pathway (such as glucose oxidase) are still very important for sensors but less so for fundamental studies, except in the special case of enzymes that use NAD(P)(H) as a mobile cofactor, which will be mentioned later.

**Fig. 3:**
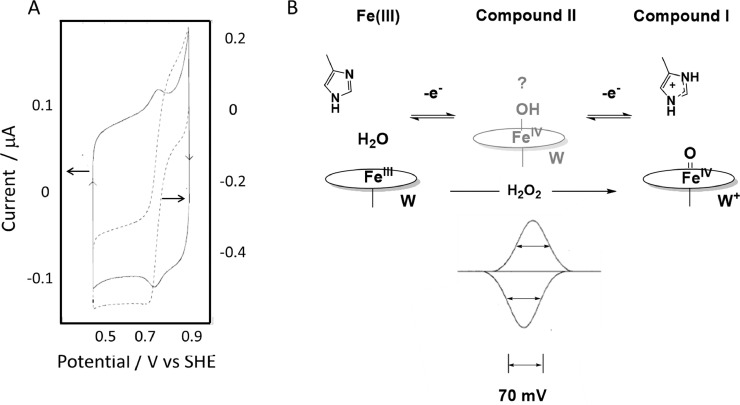
**A.** Solid line: cyclic voltammogram recorded upon introduction of a PGE electrode to a dilute (0.7 μM) solution of yeast cytochrome c peroxidase at 4 °C, pH 7. Broken line: cyclic voltammogram measured after introducing H_2_O_2_ (20 μM) Electrode rotation rate 400 rpm (not sufficiently high to overcome mass-transport limitation, as seen from the ridge at 0.7 V). **B.** Scheme showing the relationship between the non-turnover signal and the three states of the peroxidase, indicating how a proton is transferred during the ECE process. The narrow half-height widths in each direction show that the two electrons transfer with a high degree of cooperativity.

Cytochrome c peroxidase (ccp) from yeast is a Fe-porphyrin enzyme (34 kDa) which catalyses the reduction of hydrogen peroxide to water using electrons from cytochrome c. It had been noted that ccp adsorbed at a PGE electrode to catalyse H_2_O_2_ reduction in a rapid irreversible reaction commencing at around +0.8 V mV vs the Standard Hydrogen Electrode (SHE) at pH 7 [20]. By using a low temperature (4 °C) and low ionic strength (20 mM phosphate, pH 6), it was easy to see the development of a peak-like ‘non-turnover’ signal, which transformed (Fig. 3A) into a reductive catalytic wave commencing at the same potential region when H_2_O_2_ was introduced [21]. Much is already known about ccp and other peroxidases, and the catalytic interconversions are summarised in Fig. 3B [22]. During the normal catalytic cycle, hydrogen peroxide binds to the Fe(III) state of the enzyme and O—O cleavage assisted by amino acids in the distal pocket (above the Fe) leads to an intermediate, known as Compound I, which contains the ferryl group Fe(IV)=*O* and a cation radical on a nearby tryptophan (W) located on the proximal (lower) side. Compound I is returned to the Fe(III) state by two one-electron transfers from cytochrome c, via Compound II, which still contains Fe(IV)=*O* but possibly in its protonated state. Viewed instead at an electrode, the half-height widths of both oxidation and reduction peaks are each below 70 mV, showing that the two electrons transfer in a cooperative process, where the fully oxidized form must be equivalent to Compound I. A full kinetic analysis, based on the electroactive coverage measured from the non-turnover peaks showed high proficiency− turnover frequency *k*_cat_ = 268 *s* ^−^ ^1^, Michaelis constant *K*_M_ = 92 μM, *k*_cat_/*K*_M_ = 3 × 10^6^ *M* ^−^ ^1^ *s* ^−^ ^1^ at 4 °C – testiments to the high performance of a peroxidase using an unnatural redox partner (the electrode) and no mediators being required.

Following the earlier discussion of the two-electron [3Fe-4S]^0/2−^ couple that appears when a [3Fe-4S]-containing ferredoxin is adsorbed on an electrode, the cooperativity responsible for the narrow and symmetric non-turnover peaks observed for cytochrome c peroxidase thus arises also due to a reaction sequence of the ‘ECE’ type, in which an intermediary proton transfer step is needed to initiate spontaneous transfer of the second electron.

Whereas peroxidases are unidirectional enzymes, catalysing only the reduction of peroxide to water, many enzymes are bidirectional, and their electrocatalysis can be studied in both directions. Furthermore, many of these bidirectional enzymes also behave as reversible electrocatalysts, requiring only a minimal overpotential to drive a reaction in each direction [23,24]. Although it is rare to see non-turnover signals, and thus obtain absolute rates, the voltammograms convey some very special information that is never obtained in conventional enzyme kinetics. With solutions comprised of mixtures of oxidised and reduced forms of the redox couple, many adsorbed enzymes display voltammograms in which the current cuts sharply through the zero-current axis at the equilibrium potential. The obvious and essentially unique example in human technology is the reversible 2H^+^/H_2_ reaction catalysed by platinum metals. Amongst enzymes, many examples are now known, including hydrogenases, CO_2_-reducing enzymes, and mitochondrial electron-transport enzymes – all are associated with energy processes in living cells, the evolution of which has been driven by the competitive advantage of securing even small increases in efficiency [25].

**Fig. 4:**
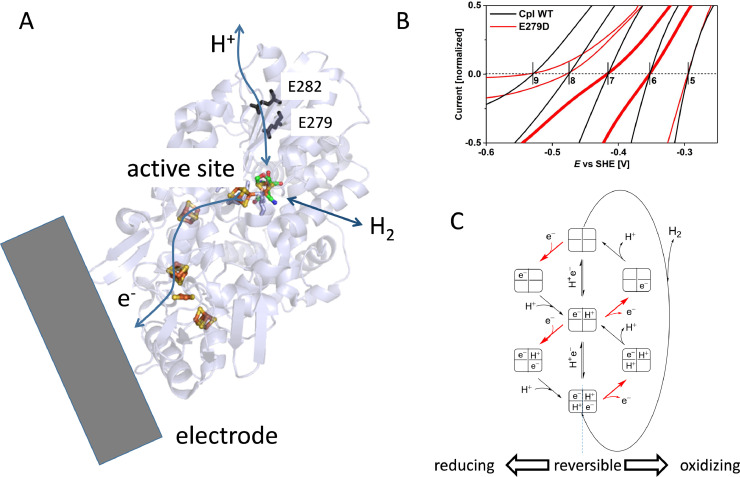
**A.** Structure of hydrogenase from *Clostridium pasteurianum* showing the manner in which electrons and protons transfer to and from the active site from opposite ends of the molecule. The enzyme must be orientated on the electrode in such a way as to allow facile electron tunnelling through the relay system of FeS clusters. The proton-transfer pathway was identified by earlier studies using site-directed mutagenesis. Two of the amino-acids in the proton-relay system are marked (glutamates 279 and 282). Changing either of these residues to aspartate (shortening the side chain by one –CH_2_- unit) has a marked effect on the catalytic voltammetry although the enzymes retain significant activity when measured by conventional solution kinetic methods. **B.** Close-up views of rotating-disc voltammograms measured for wild type and the E279D variant under 100% H_2_, in the ‘reversible’ region near to the formal potentials at different pH values. The clean ‘cut through the zero-current axis is replaced by an inflection, which is particularly marked as H_2_ evolution becomes more favoured at pH 7. **C.** Reaction pathways adopted for optimal reversibility (the vertical line combines the diagonals (concerted steps) of the two square schemes describing each half of the 2e^−^/2H^+^ = H_2_ reaction. Retarded proton transfer introduces an additional overpotential requirement in either direction by raising the cost of transferring each electron.

Evidence for the importance of *concerted* proton-coupled electron transfer (PCET) in achieving this special electrocatalytic reversibility is seen through a set of experiments on hydrogenases that could be genetically altered to disrupt proton transfer but not electron transfer [26]. As shown in Fig. 4A, a clear distinction between the two pathways was possible for certain hydrogenases for which it had been established that electrons and protons approach the buried active site from *opposite* directions – electrons using a clearly obvious relay system of FeS centres, while protons use an internal relay (determined by genetic engineering and kinetic assays) consisting of acid-base hopping sites provided by amino-acid side chains and water molecules. Lesions in the proton-transfer pathway created by mutating amino acids from E to D (shortening the sidechain by one -CH_2_- unit) caused the catalytic currents passing sharply through zero at the formal potential to take on an inflection, marking the departure from reversibility, as shown in Fig. 4B.

The appearance of inflections which can be unambiguously traced to specific structural changes in the enzyme is accounted for in terms of current models for concerted vs stepwise PCET that are based on passage of the reaction across square schemes [27,28]. The two-square scheme shown in Fig. 4C represents the step-wise and concerted transfers of electrons and protons that are required for H_2_ evolution (left and down) and H_2_ oxidation (right and up). In the ideal reversible case (the vertical line) the electrons and protons transfer in a concerted manner. Applying an overpotential in either direction accelerates electron transfer, but whereas proton transfer in the native (wild-type) enzyme is sufficiently fast to allow it to occur in concert with electron transfer, the same is not true for the mutated enzyme having a compromised proton transfer pathway. Consequently, a higher overpotential is needed to drive each electron transfer.

At this point it is worth mentioning how mediators may mask fundamental properties of redox enzymes. One interesting metric of enzymes acting as electrocatalysts when attached to an electrode is ‘catalytic bias’, the degree to which catalysis is favoured more in one direction than the other: in the case of a hydrogenase we would wish to know if and why hydrogen oxidation may be more efficient than hydrogen evolution [29]. A basic model for explaining the catalytic bias of an enzyme postulates that the bias depends on the difference between the formal potential of the reaction being catalysed (*E*_react_) and the potential of the redox centre controlling catalytic flow within the protein (*E*_ECS_); this centre is often termed the ‘electrochemical control site’ and is usually the first along an internal relay – the immediate donor/acceptor for the interfacial electrochemical step [29, 30]. The bias, expressed as the limiting current ratio *i*_ox_/*i*_red_ is given by exp{*nF*(*E*_ECS_ − *E*_react_)/*RT*); thus a bias toward oxidation occurs if *E*_ECS_ > *E*_react_ . When a mediator is used, as was described recently in a paper dealing with hydrogenase embedded in a redox polymer film, the catalytic current was greatly enhanced, which is important for designing a device: importantly, an analogous equation for bias is valid, but with *E*_ECS_ replaced by *E*_M_, the formal potential of the mediator [31]. In summary, an inherent characteristic controlling catalytic electron transfer within an enzyme is now replaced by an external factor. Other characteristics, such as alterations in reactivity accompanying specific redox transitions within an enzyme, may also be masked if the electrocatalytic current is controlled by the mediator.

## 5 Further extension to nanoconfined multi-enzyme cascade processes energized by electrochemical electron/hydride (NAD(P)(H) cycling

**Fig. 5:**
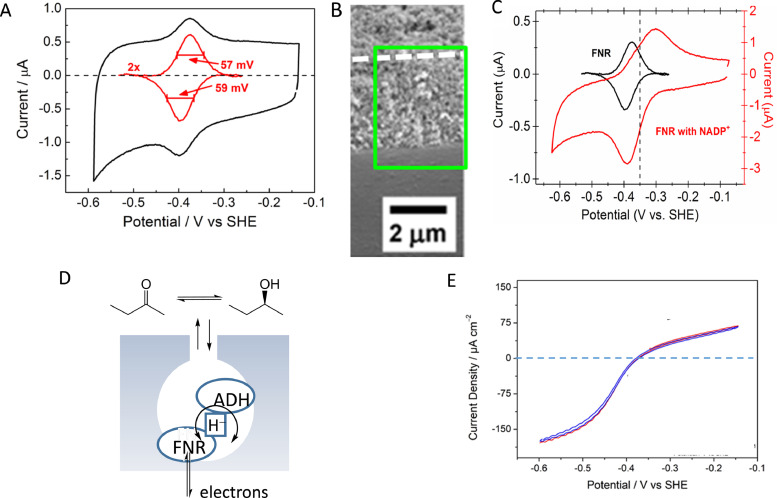
**A.** A non-turnover cyclic voltamogram measured for FNR at pH 8.0 after allowing the enzyme to adsorb from dilute solution at an ITO layer formed on a PGE electrode. **B.** SEM image of a side-on section of ITO layer formed by electrophoretic deposition of ITO nanoparticles on a ITO glass slide. **C**. Cyclic voltammogram obtained (pH 8.0) after introducing NADP^+^ to the solution; the scaled-up background-corrected non-turnover voltammogram of FNR alone is included. Broken line shows formal potential for the NADP^+^/NADPH couple. **D.** Scheme showing how FNR and a second enzyme (ADH = alcohol dehydrogenase) may be trapped in close proximity within the nanopores of the ITO layer. The nanoconfinement allows rapid recycling of NADP(H), represented here as hydride (H^−^), which couples the electrons delivered to FNR to ketone/alcohol interconversion. **E.** Cyclic voltammogram for the interconversion between 4-phenyl-2-butanone and (*S*)−4-phenyl-2-butanol present in equal concentrations at pH 9.0.

We now return to the central ‘ECE’ theme and how this transforms into ‘hydride’ transfer. In photosynthesis, electrons energized by Photosystem I are used to generate NADPH from NADP^+^, which in turn is used for anabolic reactions, particularly the fixation of CO_2_ through the Calvin cycle. The enzyme responsible, known as ferredoxin-NADP^+^ reductase (FNR), is a small monomeric enzyme, mass 39 kDa, containing a single, non-covalently bound flavin adenine dinucleotide (FAD) cofactor [32, 33]. During each cycle, two consecutive electrons are transferred from the small mediator protein, a [2Fe-2S] ferredoxin. It was discovered in 2015 that FNR is intensely electroactive at a porous indium tin oxide electrode formed by depositing ITO nanoparticles on a conducting support [34]. The resulting ITO layer behaves as a metallic conductor over the wide potential range used in the experiments. Tightly bound to the ITO, FNR displayed a sharp non-turnover signal, with half-height widths below 90 mV for both oxidation and reduction, and thus corresponding to a cooperative two-electron transfer, as shown in Fig. 5A. Significantly, electroactive coverages (reaching 500 pmol cm^−1^ at pH 9) far exceeded that expected for a monolayer, leading to the conclusion that the FNR becomes trapped in nanoscale pores that are present quite deep into the layer, the total depth of which varies between 1 and 3 μm depending on deposition time (Fig. 5B). The FAD potential was identical to that expected from earlier potentiometric studies and considerably more negative than free FAD (−0.38 V vs −0.25 V at pH 8) [34]. The pH dependence of the two-electron couple was measured and the two one-electron potentials were estimated assuming minimal dispersion. It was known already that the one-electron intermediate, necessary for facilitating the two one-electron transfers from the ferredoxin and appearing as a minority fraction during titrations, is a neutral (blue) radical, meaning that a proton is also bound [35]. Consequently, there was already an expectation that in electrochemical terms, the two-electron couple would behave as an ‘ECE’ system. Importantly, unlike in the chloroplast, the ITO electrode transfers both electrons to FNR in a single encounter, although we cannot be certain that protein dynamics allow a good deal of motion during the sequence.

The immobilised FNR is highly active catalytically. As shown in Fig. 5C, upon introduction of NADP^+^, the NADP^+^/NADPH interconversion appears as a quasi-reversible redox couple that is diffusion controlled at scan rates below 10 mV *sec*^−1^. This result is itself significant because such electrochemistry has been sought for more than half a century [33]. It was surprising to note that binding in the ITO pores not only leaves the redox properties of FNR essentially unchanged but it has also not impeded its ability to catalyse the hydride transfer reaction, which involves the binding of NADP^+^ at a critical position in relation to the FAD cofactor [34]. The central ‘ECE’ core of the electrochemical reaction corresponds to hydride transfer, the reaction that is a characteristic of nicotinamide cofactors.

An even more significant finding emerged when it was shown that binding a NADP(H)-dependant dehydrogenase as a second enzyme in the ITO nanopore network resulted in tight electrochemical coupling to that enzyme's reaction [36], [37], [38], [39]. The situation is represented in Fig. 5D, the second enzyme being an alcohol dehydrogenase that rapidly recycles the NADP(H) for immediate regeneration. The cyclic voltammetry shown in Fig. 5E is of a ketone/alcohol interconversion that is easily driven in either direction [37,38]. Consequently it is now becoming possible to carry out electrochemical studies of enzymes (extending beyond the second enzyme) that do not involve electron transfer [39]. The FNR acts as a ‘ECE’ transducer to interconvert electrons and hydride (NADPH), and the nanoconfinement of FNR with the second enzyme leads to very rapid recycling of the nicotinamide cofactor. Further enzymes, now including those that are not oxidoreductases, can be embedded in the material to achieve nanoconfined multi-enzyme cascades – the resulting situation possibly resembling that occurring in cells and organelles. Porous electrodes have a very special role to play in allowing many enzymes (and catalysts in general) to work together in cascade processes, and the natural ease by which nanoconfinement, energisation, control, and observation come together is likely to prove a winner in many areas of electrochemistry and biocatalysis. It is important to note that the term ‘nanoconfinement’ as used in this context is taken to refer to a system comprising (at least) the minimal functional enzyme pair and their coupling via nicotinamide cofactor to produce a cascade. In the case of just a single enzyme, a porous electrode offers no obvious advantage beyond increasing its surface concentration [40].

## 6 Conclusions

Confining enzymes to an electrode surface, exploiting direct electron transfer rather than using small electron mediators, reveals properties that are fundamental to the enzyme itself, most notably in the way that interfacial multi-electron transfer is tightly coupled to proton transfer within the enzyme. Curiously though, the ultimate outcome brings us back to mediators, not of electrons but of hydride, embedded and encoded in nicotinamide cofactors that have little space to move between trapped and concentrated enzyme partners.

## Declaration of Competing Interest

The author declares that he has no known competing financial interests or personal relationships that could have appeared to influence the work reported in this paper.
